# Simple and rapid determination of myristicin in human serum

**DOI:** 10.1007/s11419-012-0151-8

**Published:** 2012-08-15

**Authors:** Andrzej L. Dawidowicz, Michal P. Dybowski

**Affiliations:** Faculty of Chemistry, Maria Curie Sklodowska University, Pl. Marii Curie Sklodowskiej 3, 20-031 Lublin, Poland

**Keywords:** Myristicin, Human serum, SPE, Gas chromatography

## Abstract

Myristicin (5-allyl-1-methoxy-2,3-methylenodioxybenzene) is the main component of nutmeg (*Myristica fragrans* Houtt.) essential oil. The increasing use of myristicin as a cheap hallucinogenic intoxicant, frequently causing fatal cases of myristicin poisoning, requires new methods for determination of this compound in blood. This report describes the rapid, simple, and useful procedure for myristicin analysis in human serum, involving myristicin–protein complex degradation before chromatographic analysis. The developed method is characterized by a high recovery (above 99 %), a low detection limit (6.0 ng/g) and good repeatability (average RDS of 2.01 %).

## Introduction

Myristicin (5-allyl-1-methoxy-2,3-methylenodioxybenzene) is the main component of nutmeg essential oil [1], which exists in the nutmeg nut (*Myristica fragrans* Houtt.) and in mace, the waxy red covering of the nutmeg seed. Readily accessible on local markets, these spices have, at times, been drugs of abuse. On the other hand, nutmeg and mace are used in Asia as traditional medicines treating stomach cramps, diarrhea, and rheumatism. Information currently available on myristicin explains why its effects range from harmful to beneficial. As reported in the literature [2], this alkenylbenzene acts as a serotonin receptor antagonist and as a hallucinogenic compound. Moreover, it induces: neurotoxicity in human neuroblastoma SK-N-SH cells [3]; P450 liver enzyme in several families of rat, including the P450 1A enzyme [4], which is also induced by polyaromatic hydrocarbons, dioxins, and polychlorinated biphenyls; glutathione S-transferase [5] and forms DNA adducts in rodent livers. The ability to form DNA adducts is a common property of most chemical carcinogens and their metabolites [6]. Several tests have shown myristicin to be a weak monamine oxidase (MAO) inhibitor [7]. Acutely toxic doses of myristicin can cause organ damage [8]. Myristicin poisoning can lead to many health problems such as convulsions, delirium, blurred vision, palpitations, nausea, dehydration, general body pain, and others [9]. Those symptoms usually occur 3–6 h after the ingestion of myristicin or foodstuffs containing it, and persist up to 72 h [10]. In recent years, many cases of nutmeg poisoning have been reported, including several fatal myristicin cases [11–13]. Such poisonings can result not only from the toxic effect of myristicin itself but also from the combined toxic effects of its use with other substances [12]. Because of potential widespread human exposure through foods and beverages and the possibility of adverse effects in diverse populations, myristicin was presented to the Chemical Selection Working Group (CSWG) for review.

Two publications describing myristicin determination in biofluid can be found in the literature [14, 15]. The first concerns the myristicin metabolism in rats, but the results presented in the article were not obtained in a quantitative fashion. As a result, it is difficult to judge the method used for myristicin estimation in the reported study [14]. Moreover, the described method involves a derivatization process, which is frequently inconvenient in GC analysis (the formation of deposits in the liner of GC injector or in chromatographic column). In the second study [15], a method for HPLC determination of myristicin in rat serum is described. The procedure involves deproteinization, separation, and fractionation concentration of myristicin by appropriate switching of columns in the HPLC system. However, it should be stressed that the removal of serum proteins in the HPLC process can lead to the loss of analytes forming analyte–protein complexes. The present report describes the GC procedure for myristicin analysis in human plasma. The procedure involves a protein precipitation process, which generally degrades drug–protein complexes, and solid-phase extraction (SPE) isolation of myristicin from the examined materials. The proposed analytical approach can be considered as a method of choice for the estimation of myristicin in human fluids.

## Materials and methods

### Chemicals and reagents

Hexane and ethanol, both of analytical grade, were supplied by POCH (Gliwice, Poland). The Sepra C18-E sorbent (50 μm, 65Å) used in the SPE process was purchased from Phenomenex (Torrance, CA, USA). Myristicin, of analytical standard, was supplied by Fluka (Buchs, Switzerland). Blood for the validation procedure and blood samples containing myristicin were obtained from volunteers. Water was purified on a Milli-Q system (Millipore, Bedford, MA, USA).

### Sample preparation

#### Preparation of blood samples

Myristicin-free and myristicin-containing blood samples, obtained from healthy volunteers recruited from our laboratory, were obtained from forearm vein using Sarstedt blood collection systems. Heparin sodium was used as an anticoagulant. Myristicin was administered to the volunteers in the form of an alcoholic drink. Twenty grams of the myristicin solution (5 mg/g) was consumed by each volunteer 1 h before blood sampling.

All volunteers gave consent to participate in the studies and were made aware of the option to withdraw from further participation in the study at any time and for any reason. Each of them signed a consent form for processing of the experimental data obtained in this study.

#### Standard plasma solutions

To estimate myristicin recovery from plasma in the applied method, eight plasma samples containing 0.01, 0.1, 1, 10, 25, 50, 75, and 100 μg/g, respectively (same concentrations used for equipment calibration—see Chromatographic analysis below), were prepared. Pure plasma samples were spiked with myristicin solutions in ethanol/water mixture (30/70 % v/v). In each case, the volume of the spiking solution was less than 80 μl.

#### Myristicin isolation by SPE

To eliminate eventual loss of myristicin caused by its binding with plasma proteins, 1.5 ml of ethanol was added to 1 ml of plasma sample in a 4-ml glass vial and vortexed for 5 min. After centrifugation (945 *g* for 15 min), 1 ml of supernatant was mixed with 1.5 ml of water and 2 ml of the obtained solution was transferred into an SPE cartridge containing 0.2 g of SepraC18-E. Before loading, the cartridge was washed with 5 ml of *n*-hexane and then vacuum-dried (ca. 5 min). After removal of the loading solvent from the SPE bed, the remaining components were eluted into a calibrated flask with 2 ml of *n*-hexane and the solution was analyzed by GC. The SPE process was carried out using the SPE vacuum chamber (SPE-12G from J.T.Baker USA) at an eluent velocity of 1 drop per second.

#### Myristicin isolation by liquid–liquid extraction (LLE)

The mixture of plasma sample (1 ml) and hexane (1 ml) was vigorously shaken for 10 min at 2000 rpm. After phase separation (centrifugation), the hexane layer was subjected to GC analysis.

### Chromatographic analysis

The amount of myristicin was estimated using a GC with flame ionization detection (FID) (GC 2010, Shimadzu, Kyoto, Japan). To confirm the peak purity, each sample was also analyzed by GC-mass spectrometry (GC/MS QP2010, Shimadzu, Kyoto, Japan).

For identification of SPE extracts, a ZB5-MS fused-silica capillary column (30 m × 0.25 mm i.d., 0.25 μl film thickness; Phenomenex, USA) was used. Helium (grade 5.0) was used as carrier gas. A 1-μl aliquot of the sample was injected using an AOC-20i type autosampler. The injector’s temperature was 310 °C. The following temperature program was applied: 1 min at 50 °C, followed by a linear temperature increase up to 250 °C at 6 °C /min. The mass spectrometer was operated in EI mode at 70 eV; the ion source temperature was 220 °C. The mass spectra were measured in the range 35–360 amu. Qualitative analysis was conducted by comparing the retention indices and MS spectra for the obtained peaks with analogous data from NIST’05 to Adams databases.

Quantification of extracts was performed by injecting 1 μl of the sample using the same autosampler and capillary column. The temperature program during GC-FID separation was the same as for GC-MS.

Myristicin peak identification was carried out by comparing the GC retention index with those from GC-MS and with the retention data for myristicin standard.

The applied GC-FID equipment was calibrated using myristicin standard solutions. The working solutions were obtained by serial dilutions of the stock solution with hexane to obtain the following myristicin concentration: 0.01, 0.1, 1, 10, 25, 50, 75, and 100 μg/g.

## Results and discussion

**Fig. 1:**
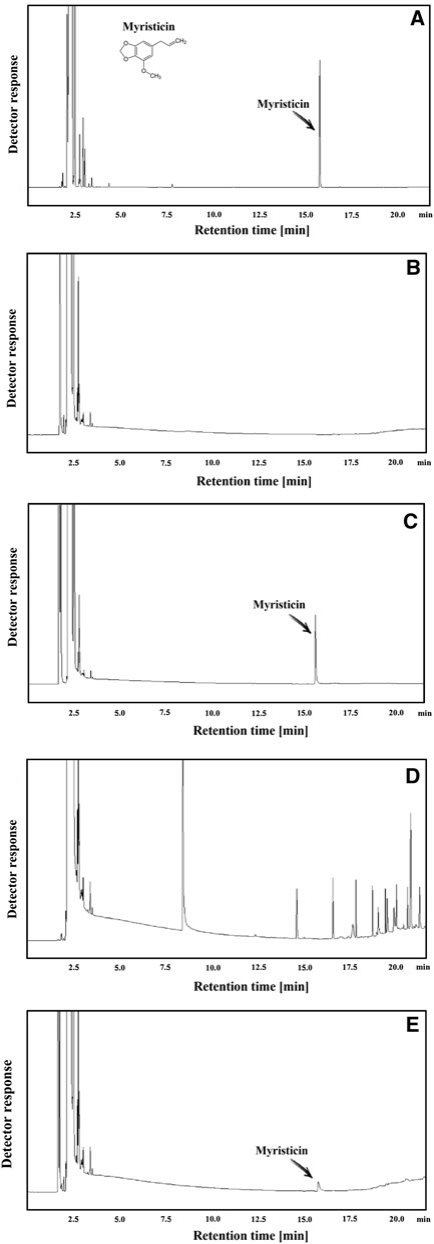
GC chromatograms of: **a** Myristicin analytical standard, **b** SPE extract from blank plasma sample, **c** SPE extract from plasma sample spiked with alcoholic solution of myristicin standard, **d** LLE extract from blank plasma, **e** SPE extract from plasma of a volunteer who consumed alcoholic solution of myristicin

Figure 1 presents chromatograms for myristicin analytical standard (Fig. 1a), SPE extracts from blank plasma (Fig. 1b), and a plasma sample spiked with alcohol solution of myristicin standard (Fig. 1c), with the two latter results were obtained according to the presented procedure. Figure 1d shows the chromatogram for the LLE extract from blank plasma, while Fig. 1e shows the additional chromatogram for the SPE extract from the plasma of a volunteer who consumed an alcohol solution of myristicin. As appears from the figure, the applied chromatographic conditions allow for sufficient resolution of myristicin from sample matrix peaks, both in the SPE and LLE procedures. However, the use of SPE seems better due to the lack of peaks occurring in the vicinity of myristicin retention in this procedure. In the case of LLE, the extract contains some cholesteric derivatives (Fig. 1d), the presence of which requires more frequent cleaning of the GC injector and leads to faster column deterioration. The identity and purity of the myristicin peak were confirmed by the MS spectra and the value of the retention index (1520), which is exactly the same as the literature value [16].

**Fig. 2:**
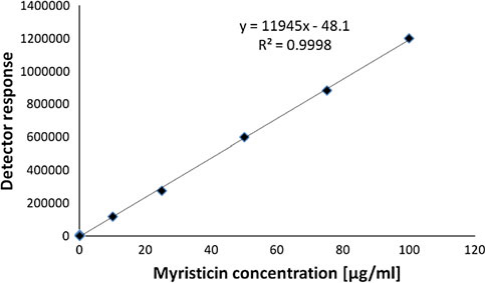
Calibration plot for myristicin: concentration range 0.01–100 μg/g

Figure 2 illustrates the calibration plot for myristicin in the concentration range from 10 ng/g to 100.0 μg/g. The resultant calibration curve shows excellent linearity with *R* ^2^ = 0.9998. The intercept value (48.1) equals 10 % of the detection limit. The obtained value exceeds the FDA requirements of *R* ^2^ ≥ 0.999 from a minimum of five observations [17, 18].

The precision and accuracy of the SPE step of the applied procedure are shown in Table 1. The presented data were obtained by five successive analyses for each sample spanning the range of the calibration curve. The examined method is characterized by very high SPE recovery (mean recovery 99.08 %) and exhibits excellent precision. The average precision of the SPE step determined as RSD for the samples at low, intermediate, and high myristicin concentrations is 2.01 %.

**Table 1:** Validation data obtained by analysis of spiked samples using the described method (*n* = 5)

Concentration (μg/g)	Myristicin		
	SD (μg/g)	RSD (%)	Recovery (%)
100	1.684	1.684	99.89
75	1.463	1.951	99.45
50	0.947	1.894	99.84
25	0.461	1.845	99.36
10	0.200	2.001	99.47
1	0.021	2.121	98.47
0.1	0.002	2.241	99.01
0.01	0.0002	2.351	97.15
Average		2.011	99.20

In order to estimate the limit of detection (LOD) and the limit of quantitation (LOQ), SPE extracts from plasma samples spiked with the myristicin standard were injected into the GC to determine the signal-to-noise (S/N) ratio. The LOD, defined as a S/N ratio of 3 was 6 ng/g, while the LOQ, defined as a S/N ratio of 10, was 20.0 ng/g.

Table 2 brings together the myristicin concentrations in human plasma samples taken from volunteers who consumed the alcoholic solution of myristicin standard 1 h before sampling. The observed differences in myristicin plasma concentration result from the different metabolisms of the volunteers connected with their age, sex, weight, etc. The estimated myristicin concentrations in human plasma are much higher than the LOQ, although the administered myristicin doses were significantly below the amount believed to cause negative symptoms in humans.

**Table 2:** Concentration of myristicin in different human serum samples (*n* = 5)

Test subject	Weight (kg)	Age (years)	Sex	Ingested dose of myristicin alcoholic solution (5 mg/g) (g)	Detected analyte (μg/g)
1	95	24	M	20	17.60
2	82	49	M	20	19.71
3	74	45	F	20	21.70
4	71	49	M	20	22.43
5	55	23	F	20	33.25

*M* male, *F* female

## Conclusions

The analytical procedure of myristicin determination in rat serum described in the literature involves deproteinization, separation, and fractionation concentration by switching of columns in the HPLC system [15]. However, it should be stressed that the removal of serum proteins in the HPLC process can lead to the loss of the analytes forming analyte–protein complexes.

The present report describes the rapid, simple, and useful procedure of myristicin analysis in human serum, involving myristicin–protein complex degradation before chromatographic analysis. The method is characterized by a high recovery (above 99 %) and a low detection limit of 6.0 ng/g, which to date are the most satisfactory values reported in the literature for myristicin determination in plasma [14, 15]. The analysis was achieved by dilution of the plasma alcoholic solution to reach a final alcohol concentration of less than 40 %. The SPE conditions for myristicin isolation from human plasma solutions agree with those elaborated for the isolation of low-molecular oxygen compounds from essential oils [19]. After slight tuning, the described method can be adapted for the determination of myristicin in other biofluids, such as cerebral spinal fluid and urine samples.
